# Safety and effectiveness of apixaban use for stroke prevention during Ramadan fasting (the API-RAM study)

**DOI:** 10.3389/fphar.2025.1565094

**Published:** 2025-09-15

**Authors:** Aiman Ghrab, Rania Gargouri, Faouzi Addad, Anis Cheikhrouhou, Mariem Jabeur, Selma Charfeddine, Amine Bahloul, Zied Triki, Tarek Ellouze, Omar Abidi, Souad Mallek, Faten Triki, Salem Abdessalem, Ismail Elalamy, Leila Abid

**Affiliations:** ^1^ Cardiology Department, Hedi Chaker University Hospital Sfax, Sfax, Tunisia; ^2^ Hannibal Clinic, Tunis, Tunisia; ^3^ Clinic Pasteur Tunis, Tunis, Tunisia; ^4^ Hematology Department, American Hospital of Paris, Paris, France; ^5^ Fondation UPM des Sciences de la Santé, Marrakesh, Morocco; ^6^ Department of Obstetrics, Gynecology and Perinatal Medicine, Sechenov University, Moscow, Russia

**Keywords:** apixaban, atrial fibrillation, Ramadan, fasting, stroke, anticoagulants

## Abstract

**Introduction:**

Patients receiving anticoagulation for atrial fibrillation (AF) are required to consult their doctor before starting Ramadan fasting to get their authorization for fasting and adapt their treatment. More often, a once-daily regimen is proposed to facilitate their intake schedule. Apixaban, a direct oral anticoagulant, prescribed twice daily with an optimal benefit/risk ratio in numerous situations, has very limited data regarding its use during Ramadan.

**Aim:**

The aim of this study was to evaluate the safety and the efficacy of apixaban for stroke prevention in AF patients during the month of Ramadan.

**Methods:**

An observational, multicentric study was performed in Tunisia during two consecutive years during the specific month of Ramadan. The API-RAM study included AF patients who were on apixaban and fasted at least 10 days. Efficacy was defined by the absence of ischemic events, and safety was established by classifying bleeding events using the BARC (Bleeding Academic Research Consortium) classification during the study period.

**Results:**

A total of 257 patients were included in our study. No ischemic events were reported during the study period. Minor bleeding events were reported in only 12 patients (4.7%), with no major bleeding event. Based on multivariate analysis, independent predictors for the bleeding risk of our population were as follows: smoking, history of hypertension, and creatinine clearance.

**Conclusion:**

Apixaban seems to be safe and effective for the prevention of the thromboembolic episode in AF patients during Ramadan fasting. Larger studies such as randomized clinical trials are necessary to confirm these results.

## 1 Introduction

Fasting is a common practice across many religions (Trabelsi et al., 2022). Within Islam, Ramadan fasting represents its last and fifth pillar, mandating Muslims to abstain from both eating and drinking throughout the days of the ninth month of the lunar calendar (Rad, 2023). The duration of fasting depends on the geographic location and the corresponding solar month and season. This seasonal variability introduces potential fluctuations in pharmacokinetics, particularly for drugs with short half-lives or multiple daily dosing regimens (Vrijens and Heidbuchel, 2015; Batarfi et al., 2021). All Muslims are obliged to fast during this month, except for those who have medical contraindication, like those with severe health problems, or who may undergo a significant risk of worse prognosis in case of fasting. Patients with cardiovascular disease are often required to visit their doctor to get his medical permission to fast (Akhtar et al., 2022). This permission may be accompanied by changes in the daily prescription, trying to simplify the treatment regimen that requires twice a day (BID, bis in die) or thrice a day (TID, ter in die) intake into a once-a-day regimen (OD). This change is done to ensure the effectiveness of the treatment during this holy month (Katada et al., 2019; Zghal Mghaieth et al., 2019). However, changing a treatment may be associated with adverse problems such as poor observance, side effects, and drug intolerance.

Direct oral anticoagulants (DOACs) have emerged as alternatives to vitamin K antagonist (VKA) therapies for stroke prevention and thrombotic complications in patients with atrial fibrillation (AF) (Bortman et al., 2023; Rosca et al., 2023). Apixaban is a DOAC, acting as an antagonist of activated factor X (FXa), taken BID. Apixaban has proven its optimal safety and effectiveness profile compared to other oral anticoagulant therapies, such as VKA and other DOACs (Granger et al., 2011). However, maintaining BID therapy during prolonged daily fasting may pose challenges in drug administration, adherence, and effectiveness, especially in vulnerable populations.

Very few studies have investigated the effect of Ramadan fasting on anticoagulation therapy (6–10). To our knowledge, no study has investigated the specific effects of fasting on patients undergoing apixaban treatment, and the impact of the BID regimen under such a unique metabolic and behavioral condition imposed by Ramadan fasting, such as dehydration, metabolic shifts, and altered gastric pH, may further complicate the pharmacokinetics and dynamics of oral anticoagulants (Vrijens and Heidbuchel, 2015; Batarfi et al., 2021).

The objective of our study is to investigate the efficacy and safety issues of BID use of apixaban for thrombosis prevention in fasting atrial fibrillation patients during the Ramadan period, aiming to establish any potential impact of fasting on the clinical outcomes of this treatment regimen.

## 2 Methods

**FIGURE 1:**
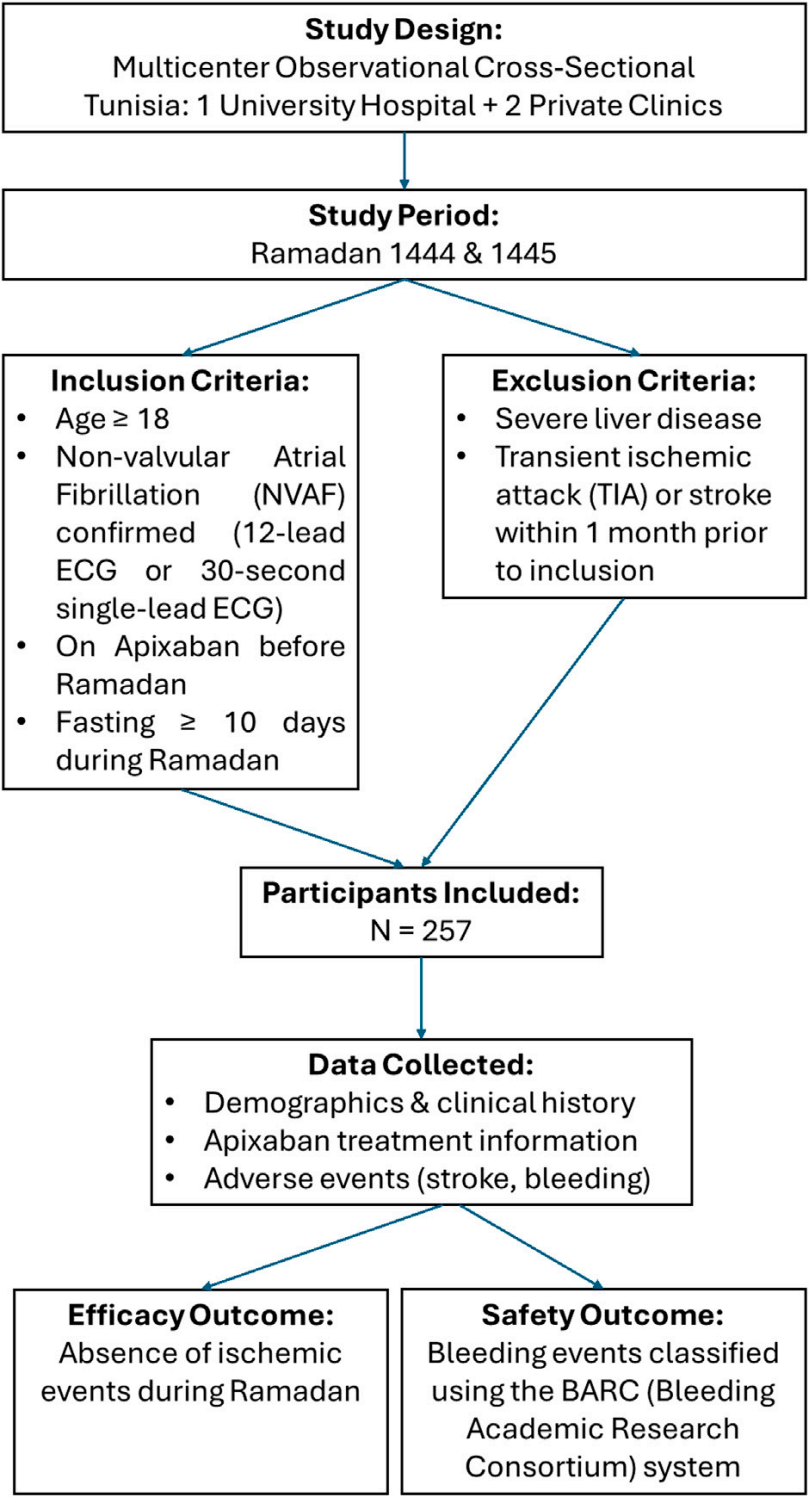
Flowchart of the API-RAM study.

We conducted a multicenter observational cross-sectional study in Tunisia at Hedi Chaker University Hospital (Pr Leila Abid, Sfax) and two private clinics (Pr. Faouzi Addad, Tunis, and Pr. Salem Abdessalem, Tunis) during Ramadan 1444 and 1445 Hijri calendar corresponding to the period spanning from March 23rd to 20 April 2023 and from March 11th to 9 April 2024. Figure 1 illustrates the flowchart of the study. Ramadan is a lunar month during which Muslims fast daily from dawn (suhoor) to sunset (iftar), abstaining from all oral intake, including food, drink, and medication. Because it follows the lunar calendar, Ramadan shifts earlier each year by approximately 11 days, resulting in seasonal variations in the duration of daily fasting, which may range from 11 to 18 h depending on the time of the year and geographic location. These fluctuations can significantly affect drug pharmacokinetics, treatment adherence, and clinical outcomes. For this reason, we included patients from two distinct Ramadan periods in two different years to enhance the robustness, reproducibility, and generalizability of our findings across various fasting durations and environmental contexts.

This study included patients under oral anticoagulant therapy who answered a survey a week after Ramadan that was created for the purpose of studying the potential impact of fasting. We included patients aged 18 years or older with non-valvular atrial fibrillation (NVAF) diagnosed and confirmed in a 12-lead ECG or 30 s of AF in a single-lead ECG recording. These patients had a documented history of NVAF for at least 1 year and had initiated apixaban therapy before Ramadan. Furthermore, patients were required to have fasted during a minimal period of 10 days during the study period. We excluded patients who had severe liver disease, transient ischemic attack, or stroke within 1 month of the inclusion date. We collected patient characteristics, apixaban therapy-related information, and adverse events. Efficacy was defined by the absence of ischemic events during the study period. Bleeding events were classified using the BARC (Bleeding Academic Research Consortium) classification system (Mehran et al., 2011). All data collected were analyzed using the Clinical Suite platform (Dacima Software), complying with the international standards, including US Food and Drug Administration 21 Code of Federal Regulations Part 11, US Health Insurance Portability and Accountability Act, International Conference on Harmonization, and Medical Dictionary for Regulatory Activities. The Clinical Suite platform allowed tracking the data entered to check for inconsistencies and missing data. All statistical analyses were performed using SPSS version 23.0 (SPSS Inc. Chicago, IL, United States) for Windows^®^.

## 3 Results

### 3.1 Study patients

During the period spanning from 23 March to 20 April 2023 and from 11 March to 9 April 2024, a total of 257 patients were included in our study. The baseline main clinical and paraclinical characteristics are summarized in Table 1. Women were more numerous, with a sex ratio of 0.7 (106 men/151 women). The median age for both sexes was 71 years. Even though a significant difference in height and weight was noted between both genders, it was found out that there was no significant difference in BMI between the sexes, that is, p = 0.208. A history of smoking was higher in male patients (34%) than in female patients (20.5%) (p = 0. 016), and active smoking had higher significant difference (p < 0.001). In this study, hypertension was significantly more prevalent among female participants (76.2%) than among male participants (64.2%) (p = 0.036). Chronic kidney disease was more frequent and severe in men highlighted by a significantly lower creatinine clearance (61.7 mL/min vs. 75.7 mL/min, p < 0.001), with a higher proportion of men having an impaired creatinine clearance <60 mL/min (48% vs. 25%, p = 0.001). No patient had a severe renal insufficiency at the inclusion.

**TABLE 1:** Baseline characteristics and sex difference of the study population.

Variable	Male (n = 106)	Female (n = 151)	Total (n = 257)	p
Age, median [min; max]	71 [28; 88]	71 [44; 86]	71 [28; 88]	0.25
Weight (kg), mean ± SD	82.2±14	78.8±10.5	77.7±12.6	**0.004**
Height (cm), mean ± SD	173±7.6	164.6±7.5	167.8±8.6	**<0.001**
BMI	27.6±6.7	28.3±4.4	28±-4.4	0.208
History of smoking, n (%)	36 (34)	31 (20.5)	67 (26.1)	**0.016**
Active smoking, n (%)	17 (16)	3 (2)	20 (7.8)	**<0.001**
Alcohol, n (%)	4 (3.8)	0	4 (1.6)	**0.028**
Diabetes mellitus, n (%)	30 (28.3)	51 (33.8)	81 (31.5)	0.353
Type 2 diabetes, n (%)	30 (28.3)	51 (33.8)	81 (31.5)	0.353
Hypertension, n (%)	68 (64.2)	115 (76.2)	183 (71.2)	**0.036**
Dyslipidemia, n (%)	45 (45.5)	61 (40.4)	106 (41.2)	0.742
Active cancer, n (%)	1 (0.9)	1 (0.7)	2 (0.8)	1
Pacemaker, n (%)	6 (7.1)	0	6 (3.1)	**0.006**
Heart failure, n (%)	49 (46.2)	62 (41.1)	111 (43.2)	0.41
COPD, n (%)	2 (1.9)	1 (0.7)	3 (1.2)	0.571
Sleep apnea, n (%)	10 (9.4)	22 (14.6)	32 (12.5)	0.22
Stroke, n (%)	16 (15.1)	23 (15.2)	39 (15.2)	0.976
AF ablation, n (%)	7 (8.3)	2 (1.8)	9 (4.6)	**0.042**
Ischemic heart disease, n (%)	11 (10.4)	30 (19.9)	41 (15.9)	**0.023**
Anterior PCI, n (%)	10 (9.4)	19 (12.6)	29 (11.3)	0.588
Anterior CABG, n (%)	0	1 (0.7)	1 (0.4)	0.401
Creatinine clearance	61.7 [31.1; 92.3]	75.7 [39.7; 106.7]	72 [39; 105]	**<0.001**
Creatinine clearance <60 mL/mn	36/75 (48%)	32/128 (25%)	68/203 (33.5%)	**0.001**
HB, mean ±SD	13.7±1.5	12.8±1.4	13.2±1.5	**<0.001**
Heart rate on ECG, median [Q1, Q3]	68 [60.77]	70 [63.84]	69 [61.83]	0.073
AF on ECG, n (%)	45 (42.5)	64 (42.4)	109 (42.4)	0.991
CHA2DS2-VA	3.35±1.6	3.1±1.4	3.21±1.46	0.203
HAS-BLED	1.77±0.8	1.75±0.9	1.76±0, 9	0.396
LVEF	62.2±9	61.9±8.9	61.9±9	0.644
LA surface	23±7.2	22.3±4.8	22.5±5.7	0.874
LA diameter	43±10.7	43±6.1	43±7.9	0.848
Antiarrhythmics, n (%)	42 (39.6)	48 (32)	90 (35.2)	0.208
ARB, n (%)	23 (21.7)	33 (21.9)	56 (21.8)	0.976
ACEi, n (%)	21 (19.8)	40 (26.5)	61 (23.7)	0.215
Diuretics, n (%)	39 (36.8)	57 (37.7)	96 (37.4)	0.876
Beta-blockers, n (%)	66 (62.3)	94 (62.3)	160 (62.3)	0.998
Calcium channel blockers, n (%)	6 (5.7)	13 (8.6)	19 (7.4)	0.374
SGLT2i, n (%)	10 (9.4)	21 (13.9)	31 (12.1)	0.278
Statins, n (%)	45 (42.5)	67 (44.4)	112 (43.6)	0.76
Nonsteroidal anti-inflammatory drugs, n (%)	1 (0.9)	3 (2)	4 (1.6)	0.645

Bold values indicate statistically significant results (p < 0.05).

The mean CHA2DS2-VA score was 3.21±1.46, and the mean HAS-BLED score was 1.76±0.9. A total of 143 patients had paroxysmal NVAF (55.6%), and the remaining patients had either persistent or permanent AF.

The great majority of the study population got the fasting authorization par their doctors (n = 210, 82%).

### 3.2 Study outcomes

All patients received either 5 mg BID or 2.5 mg BID of apixaban according to the guidelines: low-dose apixaban (2.5 mg BID) was used based on the following standard clinical criteria: age ≥80 years, body weight ≤60 kg, or serum creatinine ≥1.5 mg/dL, as per guideline recommendations. A total of 57 patients (22.2%) reported missing at least one dose of apixaban during the study period. Any ischemic event was reported during the study period.

Minor bleeding events were reported by 12 patients (4.7%): four cases of gingivorrhagia, five incidents of bruising, two minor nosebleeds, and one case of gastrointestinal bleeding secondary to hemorrhoids. No major bleeding event was reported.

Table 2 illustrates the result of bivariate analysis of factors associated with the occurrence of bleeding events. Impaired renal function evaluated by creatinine clearance was the most important predicting factor associated with bleeding complications. This group of patients had significantly lower creatinine clearance than patients without bleeding events (43.77 ± 10.8 mL/min vs. 76.3 ± 26.9 mL/min; p-value < 0.001). Active smoking was more prevalent in the bleeding event group. However, having a history of smoking, whether a current or former smoker, was significantly higher in patients with a bleeding episode (58.3% vs. 24.5%; p = 0.016). Interestingly, patients treated for hypertension were less likely to have bleeding complications (72.7% vs. 41.7%; p = 0.043). The other cardiovascular risk factors did not significantly differ between the two groups.

**TABLE 2:** Analysis of factors associated with bleeding events.

Variable	No bleeding event (n = 245)	Bleeding event (n = 12)	p-value
Age, median [min; max]	69.3 (28; 88)	70.2 (52; 86)	0.884
Sex, n (%)			
Male	103 (42)	3 (25)	0.369
Female	142 (58)	9 (75)	
Weight (kg), mean ± SD	77.8 ± 12.5	75 ± 16.1	0.377
Height (cm), mean ± SD	167.9 ± 8.6	166.3 ± 7.4	0.599
BMI	28 ± 6.4	27.5 ± 4.7	0.89
Active smoking, n (%)	18 (7.3)	2 (16.7)	0.237
History of smoking, n (%)	60 (24.5)	7 (58.3)	**0.016**
Diabetes mellitus, n (%)	76 (31)	5 (41.7)	0.438
Hypertension, n (%)	178 (72.7)	5 (41.7)	**0.043**
Dyslipidemia, n (%)	102 (41.6)	4 (33.3)	0.766
Cancer history, n (%)	2 (0.8)	0	1
Pacemaker, n (%)	5 (2.7)	1 (9.1)	0.299
Heart failure, n (%)	104 (42.4)	7 (58.3)	0.278
COPD, n (%)	2 (0.8)	1 (8.3)	0.134
Sleep apnea, n (%)	30 (12.2)	2 (16.7)	0.65
Stroke, n (%)	37 (15.1)	2 (16.7)	1
AF ablation, n (%)	9 (4.9)	0	1
Ischemic heart disease, n (%)	39 (15.9)	1 (8.3)	0.698
Anterior PCI, n (%)	29 (11.8)	1 (8.3)	1
Anterior bleeding event, n (%)	26 (10.6)	1 (8.3)	1
Anterior GI bleeding, n (%)	3 (1.2)	1 (8.3)	0.175
Anterior cerebral bleeding, n (%)	3 (1.2)	0	1
Anterior major bleeding, n (%)	4 (1.6)	0	1
Anterior minor bleeding, n (%)	25 (10.2)	0	0.614
Creatinine clearance, mean ± SD	76.3 ± 26.9	43.77 ± 10.8	**<0.001**
HB, mean ± SD	13.2 ± 1.4	13.1 ± 1.7	0.761
Heart rate on ECG, median [Q1, Q3]	72 ± 14, 9	80.8 ± 20.8	0.378
AF on ECG, n (%)	106 (43.3)	3 (25)	0.247
CHA2DS2-VA	3.18 ± 1.45	3.66 ± 1.55	0.147
HAS-BLED	1.76 ± 0.9	1.83 ± 0.9	0.658
LVEF	62 ± 9.12	61.8 ± 5.5	0.722
LA surface	22.5 ± 5.8	22.6 ± 0.9	0.522
LA diameter	42.8 ± 8	44.8 ± 1.78	0.386
Antiarrhythmics, n (%)	86 (35.2)	4 (33.3)	1
ARB, n (%)	53 (21.6)	3 (25)	0.728
ACEi, n (%)	56 (22.9)	5 (41.7)	0.163
Diuretics, n (%)	92 (37.6)	4 (33.3)	1
Beta-blockers, n (%)	152 (62)	8 (66.7)	1
Calcium channel blockers, n (%)	16 (6.5)	3 (25)	**0.049**
SGLT2i, n (%)	29 (11.8)	2 (16.7)	0.643
Statins, n (%)	105 (42.9)	7 (58.3)	0.375
Nonsteroidal anti-inflammatory drugs, n (%)	4 (1.6)	0	1
Fasting authorization, n (%)	200 (81.6)	10 (83.3)	1
Short interval between intakes	8 h [7 h; 9 h]	9 h [8 h; 10 h]	0.271
Long interval between intakes	16 h [15 h; 17 h]	15 h [14 h; 16 h]	0.278
Missed dose, n (%)	52 (21.2)	5 (41.7)	**0.146**
Number of fasting days	29.5	28.33	**0.46**

Patients who reported having a bleeding event had a higher CHA2DS2VA score (3.66±1.55 vs. 3.18±1.45, p = 0.147) but a similar HAS-BLED score (1.76±0.9 vs. 1.83±0.9; p = 0.658).

Surprisingly, not having the physician’s authorization did not increase the risk of bleeding [n = 10/210 (4.7%) vs. n = 2/47 (4.25%)] or thrombosis. Moreover, bleeding events were more often observed in patients who reported having missed at least one dose intake of apixaban (22.2%), but without reaching a statistically significant difference [n = 52 (21.2%) vs. n = 5 (41.7%); p = 0.146]. A comparative analysis of clinical and demographic characteristics between patients who reported forgetting to take their anticoagulant medication (non-adherent group, n = 57) and those who maintained adherence (adherent group, n = 200) is presented in Table 3. Overall, no significant differences were observed across most variables. However, previous history of percutaneous coronary intervention (PCI) and the use of angiotensin receptor blockers (ARBs) were significantly more frequent in the adherent group (p = 0.012 and p = 0.020, respectively). A trend toward higher rates of bleeding events was noted among non-adherent patients; however, it did not reach statistical significance. These findings may suggest that a more complex or advanced cardiovascular history could influence adherence behaviors or tolerability of the anticoagulant regimen.

**TABLE 3:** Comparison of patient characteristics by medication adherence.

Characteristic	Non-adherent group (n = 57)	Adherent group (n = 200)	P-value
Sex (male), n (%)	28 (49.1)	78 (39.0)	0.171
Smoking history (yes), n (%)	19 (33.3)	48 (24.0)	0.157
Active smoking (yes), n (%)	5 (8.8)	15 (7.5)	0.752
Alcohol consumption (yes), n (%)	2 (3.5)	2 (1.0)	0.177
Diabetes (yes), n (%)	19 (33.3)	62 (31.0)	0.738
Hypertension (yes), n (%)	37 (64.9)	146 (73.0)	0.234
Dyslipidemia (yes), n (%)	26 (45.6)	80 (40.0)	0.448
Neoplasia (yes), n (%)	1 (1.8)	1 (0.5)	0.342
Pacemaker (yes), n (%)	3 (6.3)	3 (2.1)	0.145
Congestive HF (yes), n (%)	22 (38.6)	89 (44.5)	0.427
Chronic kidney disease (yes), n (%)	1 (1.8)	2 (1.0)	0.64
Anterior PCI (yes), n (%)	12 (21.1)	18 (9.0)	0.012
ARB (yes), n (%)	6 (10.5)	50 (25.0)	0.02
Antiarrhythmic use (yes), n (%)	26 (45.6)	64 (32.2)	0.061
Diuretics (yes), n (%)	21 (36.8)	75 (37.5)	0.928
Calcium channel blockers (yes), n (%)	2 (3.5)	17 (8.5)	0.204
Beta-blockers (yes), n (%)	37 (64.9)	123 (61.5)	0.639
Statins (yes), n (%)	25 (43.9)	87 (43.5)	0.961
Fixed drug association (yes), n (%)	25 (43.9)	86 (43.0)	0.908
Bleeding event, n (%)	5 (8.8)	7 (3.5)	0.096

**FIGURE 2:**
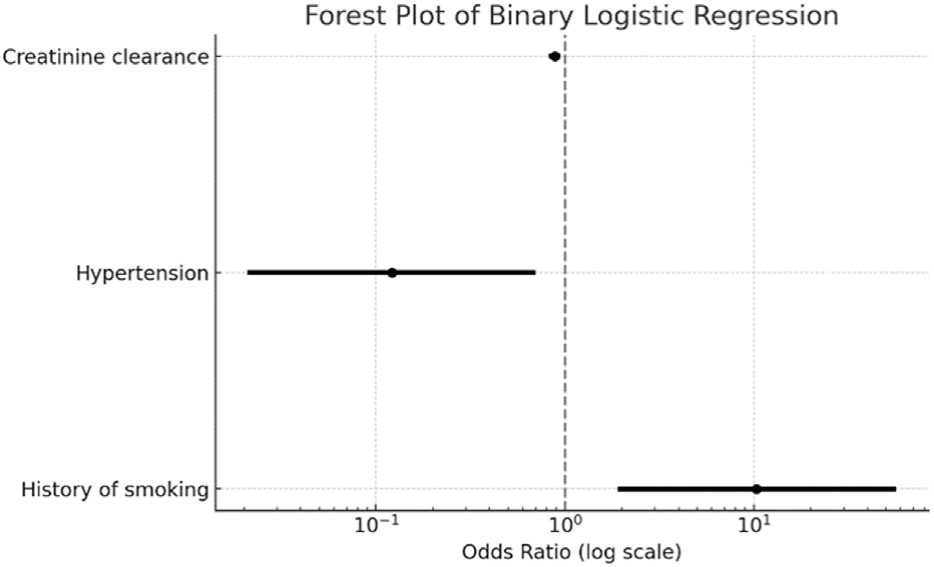
Forest plot of binary logistic regression analysis fro bleeding events. The figure displays the odds ratios (log scale) with 95% confidence intervals for predictors of bleeding events in the API-RAM study. Variables included in the model were creatinine clearance, hypertension, and history of smoking.

Based on multivariate analysis, independent predictors of bleeding risk of our population were smoking, history of hypertension, and creatinine clearance. Table 4 and Figure 2 illustrate the interaction of these parameters with bleeding events.

**TABLE 4:** Independent predictors of bleeding risk.

Parameter	OR	CI	p-value
History of smoking	10.342	(1.894; 56.48)	0.007
Hypertension	0.122	(0.021; 0.703)	0.019
Creatinine clearance	0.883	(0.823; 0.947)	0.001

**FIGURE 3:**
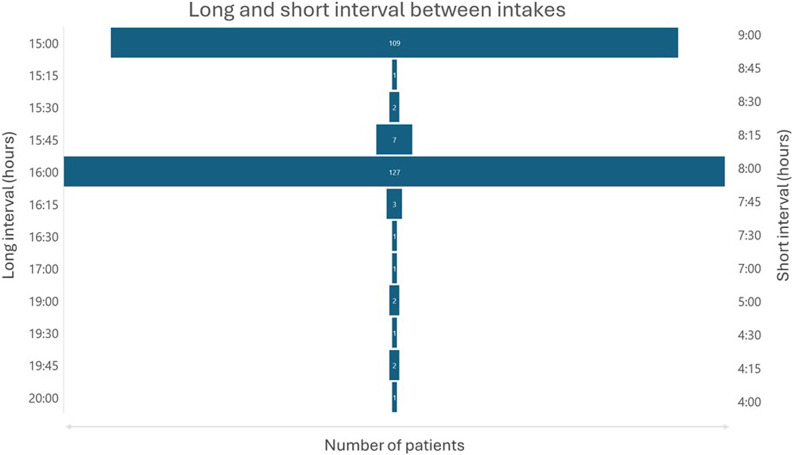
Distribution of morning and evening dose administration times. The histograms illustrate the timing of anticoagulant intake in the API-RAM study.

**FIGURE 4:**
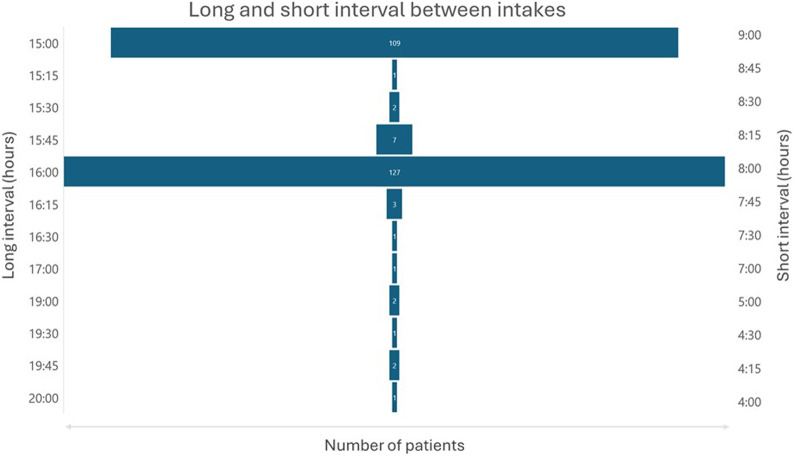
Long and short intervals between morning and evening doses.

Most patients had their evening dose intake during Iftar or shortly after, and their anteriorly morning dose intake during Sahour is as illustrated in Figure 3. The average duration of the short interval between doses was 8 h and 20 min with extremes ranging from 4 h to 9 h. The average duration of the long interval between doses was 15 h and 38 min with extremes ranging from 15 h to 20 h as shown in Figure 4. The duration interval between drug intakes affect neither the bleeding risk nor the thrombotic risk (p = 0.271).

## 4 Discussion

Our study aimed to evaluate the safety and efficacy of apixaban in patients with NVAF during Ramadan fasting. The results provide reassuring insight into the safety profile, adherence, and efficacy of apixaban during this period of religious fasting. Even though 22.2% have missed at least one dose of apixaban, it did not affect the treatment efficacy during the study period. To have the best time interval between treatment intakes (Anghel et al., 2019; Yilmaz, 2023), patients were required to wake up every night during Sahour, the period before dawn, to take their pill, which was not an easy task, especially for old adults with multiple comorbidities, as in our study population. However, they were already advised to wake up to drink reasonably and limit the risk of dehydration during their fasting day. Taking in consideration all these variables, we can conclude that the apixaban BID regimen had a satisfactory adherence in our population as the majority had their first dose during or shortly after Iftar and their second dose during Sahour or slightly before Imsak.

Despite all the challenges the fasting poses, which could induce a hypercoagulable state with dehydration (Almulhem et al., 2020), decreased blood sugar levels (Gajos et al., 2015), increased stress hormones (Sandrini et al., 2020; Kim et al., 2021), reduced mobility due to fatigue, and the disruption of the circadian rhythm (Peñaloza-Martínez et al., 2022), apixaban succeeded in protecting its users from stroke and ischemic events during the Ramadan fasting period. These findings reinforce the robustness of the anticoagulant effect of this drug in such a particular period with a potential aggravation of the patient prothrombotic profile.

Numerous studies proved that intermittent fasting enhances the overall health by improving the cardiovascular functioning, controlling diabetes, and reducing the incidence of cancer and neurological disorders (Kim et al., 2021). Intermittent fasting during Ramadan may be even more beneficial attention as it is also associated with better mental status and less stress due to the spiritual energy gained while practicing prayers and religious activities. These activities guarantee patient mobility, reducing thrombotic risk related to an eventual stasis that may be associated with bed rest often described during intermittent fasting due to fatigue. A recent study has shown that Ramadan intermittent fasting was associated with an increased physiological anticoagulant activity through an augmented production of protein S in the fasting patient group (Al-Ghumlas, 2024). Therefore, the absence of thrombotic complication in our study may be due not only to the protective effect of the molecule but also to the enhanced innate anticoagulant effect during this holy month.

Only 4.7% of patients experienced minor bleeding episodes, which included nosebleeds, bruises, gingivorrhagia, and hemorrhoid bleeding. These minor bleeding episodes were easily controllable and did not require discontinuation of the apixaban use. This observation proves that apixaban preserves a good safety profile in patients who are fasting during Ramadan and which is similar to the tolerance observed in non-fasting patients (Agrawal et al., 2024).

Moderate impaired kidney function was an independent predictor of bleeding complication in our study as reported in the literature. Taking these findings into consideration, we recommend carefully monitoring renal function in this group of patients with renal insufficiency as fasting increases the risk of dehydration that could significantly affect the kidney function and reduce the creatinine clearance; dose adjustment may be needed to treat severe functional renal dysfunction through rehydration or by interrupting Ramadan fasting to prevent other potential complications. In a recent study, there was a higher risk of bleeding associated with full-dose apixaban versus half-dose in patients with atrial fibrillation and severe chronic kidney disease, but no difference was reported in stroke/systemic embolism or death occurrence (Xu et al., 2023).

Currently or formerly smoking was also an independent predictor of bleeding events. Smoking causes atherosclerosis and damages the vascular walls, resulting in vessel’s fragility increasing the risk of bleeding, particularly in those on anticoagulation therapy. This suggests that smoking cessation can be a valuable intervention which could reduce not only thrombotic but also bleeding risk in this population of patients. Our findings are consistent with a previously published study that studied the effect of smoking on the bleeding risk (Langsted and Nordestgaard, 2018).

Interestingly, patients treated for hypertension were less prone to bleeding complication. However, most clinical evidence suggests that hypertension increases the risk of bleeding in patients receiving anticoagulation (Shoeb and Fang, 2013; Harskamp et al., 2022). This may reflect the presence of undiagnosed masked hypertension in patients who had bleeding events as being not treated exposes them to arterial pressure fluctuations that may cause minor bleedings. These findings suggest investigating masked hypertension with potential treatment resistance through regular auto-monitoring of patient arterial pressure, instead of discontinuing or changing the anticoagulation regimen.

Patients in the bleeding event group were less compliant as they missed medication doses more than the non-bleeding group. This is underlying the fact that the bleeding risk is not only related to anticoagulation but much more to patient vulnerability. Non-compliance to oral anticoagulation therapy during Ramadan setting can be caused mainly by missing the chance to get up early in the Sahour before the Imsak time (Mirghani et al., 2023). As observed in our study, having missed a dose was not exposing the patient to a higher thrombotic risk, but it may be associated with certain mistaken behaviors such as taking a double dose during the next dose’s time. Moreover, some patients did not respect the minimal recommended 6 h interval between the doses of apixaban (AlShoaibi et al., 2021). Therefore, this non-compliance may cause fluctuations in apixaban plasma concentrations, which can facilitate the bleeding risk. This underlines the need of an efficient therapeutic education before this holy month to avoid or limit such a bleeding complication in these fasting patients. In a previous study (Batarfi et al., 2021), patients on a BID anticoagulation treatment had three times higher risk of hospitalization. However, this was mainly due to the high prevalence of self-guided treatment regimen modification in this group of patients who changed their intakes without consulting their physician, a wrong attitude leading to more dramatic consequences. Reviewing the published studies about anticoagulation during Ramadan intermittent fasting, we can conclude the optimal clinical benefit of DOACs compared to VKA. Addad et al. (2014) and Sridharan et al. (2021) proved that VKA usage led the patients to a higher bleeding risk as the INR was more prone to a significant increase during this period and especially in the first 2 weeks. On the other hand, AlAyoubi (2021) studied the effect of Ramadan on two DOACs, rivaroxaban and dabigatran, and have shown their better safety and efficacy profile during intermittent fasting. Our study reinforces the choice of DOACs for oral anticoagulation during Ramadan.

On the other hand, AlAyoubi (2021), in a study held in Saudi Arabia during the month of Ramadan 1441 Hijri (June 2019), has evaluated the efficacy and tolerance of NOACs, whether taken once (rivaroxaban) or twice (dabigatran) daily during the fasting period of Ramadan, for the prevention of ischemic events in patients with non-valvular atrial fibrillation. The study included 114 patients who fasted for at least 15 days. Its aim was to compare the incidence of clinical events 1 month before Ramadan (non-fasting period) and during Ramadan (fasting period). Despite different fasting conditions (socio-cultural context, climate, and fasting hours) and the use of a different molecule (dabigatran), fasting during Ramadan was not associated with an increased risk of ischemic or hemorrhagic events in patients treated with NOACs on a twice-daily regimen and did not result in significant changes in hemostasis or kidney function parameters (p > 0.05).

Once-daily dosing is often considered better for adherence. During Ramadan, rivaroxaban may be perceived as more appropriate than apixaban due to its 24-h coverage and single daily dose, which may reduce fasting-related risks. However, this view mainly focuses on dose completion rates, not on correct timing of intake. In fact, medication effectiveness depends not only on how many doses are taken but also when they are taken. Irregular or poorly timed dosing can reduce treatment effectiveness, even with high adherence rates. Proper risk assessment should include both dose timing and how it affects the drug’s behavior in the body, ideally using electronic monitoring. For example, a simulation study (Vrijens and Heidbuchel, 2015) has shown that twice-daily NOAC regimens offer more stable drug levels, are less sensitive to missed or extra doses, and carry a lower risk of extreme concentration changes than once-daily regimens, particularly that a single missed once-daily dose is more impactful than missing one dose in a twice-daily schedule. Our study joins its predecessors in strengthening the place of using NOACs for stroke prevention during Ramadan. However, according to real-world clinical findings, such as the Saudi study by Batarfi et al. (2021), suboptimal medication adherence is a widespread problem in ambulatory care of chronic diseases, with deviations in either direction from the prescribed dosing regimen. For the NOACs, such deviations occur and can lead to bleeding or clotting as suboptimal adherence involves temporary periods of either overdosing or underdosing. In this expert review, we discuss the following: (a) the proper definition of adherence in terms of its three elements: initiation, implementation, and discontinuation; (b) how adherence is reliably and accurately measured; and (c) how adherence is successfully enhanced, to achieve and maintain safe and effective levels of NOAC-based anticoagulation. We also discuss the comparative effects of prescribing the same total daily dose, given either once-daily or as half-strength twice-daily doses. Because NOACs have plasma half-lives of ∼12 h, the twice-daily dosing regimen is less prone than the once-daily dosing regimen to hazardously high peaks or hazardously low troughs in anticoagulant concentrations and associated actions. In other fields of oral drug treatment, the continuity of drug action is greater with twice-daily than with once-daily dosing; however, a few more doses are skipped with twice-daily than with once-daily dosing. This paradox is explained by the disproportionately greater impact on drug action of skipping a once-daily than a twice-daily dose. Integration of these principles into real-world medication management is the next step in the improvement of oral anticoagulation (Batarfi et al., 2021), which raises concerns about adherence issues with twice-daily NOAC use during Ramadan. This study consists of 808 fasting patients on anticoagulants during Ramadan, finding that over half (53.1%) adjusted their medication schedule, with more changes observed in those on twice-daily regimens and with lower education levels. These modifications were linked to higher hospitalization risk, although not directly to dosing frequency. However, the study’s results are limited by non-standard dosing practices, heterogeneous patient groups, and nonspecific hospitalization causes, making it unclear whether all schedule changes were clinically harmful; some modifications may even have been simple harmless adjustments for fasting time.

Current guidelines that recommend changing a BID anticoagulation regimen into a QD regimen as mentioned in the latest Tunisian National Institute for Health Evaluation and Accreditation (INEAS) are questionable. They recommend prescribing rivaroxaban during Iftar instead of using apixaban as BID regimen may expose the patients to side effects due to the non-respect of the 12 h interval based on the study mentioned above (Batarfi et al., 2021). We insist that the clinical study has only shown that these patients with a self-guided treatment modification and insufficient therapeutic education were at higher risk for complications. In fact, there is no proven clinical impact of such a delayed interval. Moreover, in the apixaban monography, it is allowed to miss a pill intake without any change or to take the forgotten one before a delay of 6 h after the planned time, thus leading to reach a maximal interval of 18 h between the two pill intakes. Therefore, both the large interval of the protective effect and the confirmed comfort of the clinical tolerance are respected whatever the fasting period duration is. Our study is reinforcing this added value of apixaban with an optimal clinical benefit in this vulnerable population, which requires the less harming and the most protecting strategy against cardiovascular risks.

Patients included in our study were older, with more comorbidities, and had higher CHA2DS2VAsc and HAS-BLED scores than the general atrial fibrillation population, as shown in the Tunisian national registry NATURE-AF (Ouali et al., 2021), proving that our population had baseline significantly higher thrombotic and bleeding risks.

The main limitations of our study are its observational design and its relatively small population. However, our population had patients from the same country, thus having the same fasting hours. This may affect the applicability of our findings on other populations or the same population with different fasting hours. Future research should be conducted using larger populations with different fasting hours during a larger time period to cover more than a Ramadan month.

## 5 Conclusion

Our study focused on the efficacy and safety profile of apixaban BID as anticoagulation therapy for antithrombotic prevention in patients with NVAF during Ramadan fasting. We provided valuable insights to the medical community and the Muslim society since this observational and prospective study is one of the very few studies on anticoagulation treatment during fasting showing the optimal clinical benefit of apixaban in these particular circumstances. These encouraging data require further studies to enlarge the magnitude of this strategy in various Ramadan seasons and regions.

## Data Availability

The raw data supporting the conclusions of this article will be made available by the authors, without undue reservation.
