# Supplementary material for: Safety and effectiveness of apixaban use for stroke prevention during Ramadan fasting (the API-RAM study)
Source: Front Pharmacol. 2025 Sep 15;16:1565094. doi: 10.3389/fphar.2025.1565094 (PMC12476992; doi:10.3389/fphar.2025.1565094)
Supplement: Supplementary file 1 [file DataSheet1.pdf]

Supplementary Material

1 Supplementary Data

Appendix A. Follow-Up Survey Form (Post-Ramadan Telephone Interview)

Contact and Follow-Up

• Was the patient reachable by phone? ☐ Yes ☐ No

• Date of the telephone interview: ___ / ___ / ______ (DD/MM/YYYY)

Treatment Continuity

• Did the patient continue taking Apixaban during Ramadan? ☐ Yes ☐ No

• If no, reason for discontinuation or change:

• ____

Fasting Information

• Did the patient fast during the month of Ramadan? ☐ Yes ☐ No

• Reason/motivation for fasting:

☐ Physician's authorization

☐ Personal/spontaneous decision

• Number of days the patient fasted: __________ days

• Time of first dose intake (during Ramadan): __ : __ (24-hour format)

• Time of second dose intake (during Ramadan): __ : __ (24-hour format)

• Did the patient miss one or more doses of Apixaban during Ramadan? ☐ Yes ☐ No

• Comments (if any):

• ____

Clinical Events During Ramadan

• Did the patient experience any clinical event? ☐ Yes ☐ No

Hospitalization

• Was the patient hospitalized? ☐ Yes ☐ No

• Date of hospitalization: ___ / ___ / ______ (DD/MM/YYYY)

• Reason for hospitalization:

• ____

Thromboembolic Event

• Did the patient experience a thromboembolic event? ☐ Yes ☐ No

If yes, specify:

• Systemic embolism: ☐ Yes ☐ No

• Transient or permanent event: ☐ Transient ☐ Established

• Cerebral event (e.g., stroke): ☐ Yes ☐ No

• Date of the thromboembolic event: ___ / ___ / ______ (DD/MM/YYYY)

Hemorrhagic Event

• Did the patient experience a bleeding event? ☐ Yes ☐ No

If yes, specify the origin:

☐ Digestive ☐ Cerebral ☐ Other: ____________________________

• Date of the hemorrhagic event: ___ / ___ / ______ (DD/MM/YYYY)

Definition of Major Bleeding:

A major bleeding event is defined as:

- Drop in hemoglobin ≥ 2 g/dL, OR

- Transfusion of ≥ 2 units of packed red blood cells or whole blood, OR

- Bleeding in a critical site (intracranial, intraspinal, intraocular, intra-articular, pericardial, retroperitoneal, or intramuscular with compartment syndrome), OR

- Fatal bleeding

• Was the bleeding event considered major? ☐ Yes ☐ No

• Date of major bleeding: ___ / ___ / ______

• Was the bleeding event considered minor? ☐ Yes ☐ No

• Date of minor bleeding: ___ / ___ / ______

Other Adverse Events

• Specify any other adverse effects:

• ____

Death

• Did the patient die during or after Ramadan? ☐ Yes ☐ No

• Date of death: ___ / ___ / ______

• Cause of death:

☐ Cardiovascular origin ☐ Other: _________________________

- If cardiovascular, specify: ______________________________

- If other, specify: ______________________________
